# Supplementary material for: The Cocrystal of Ubiquinol: Improved Stability and Bioavailability
Source: Pharmaceutics. 2023 Oct 20;15(10):2499. doi: 10.3390/pharmaceutics15102499 (PMC10610044; doi:10.3390/pharmaceutics15102499)
Supplement: Supplementary file 1 [file pharmaceutics-15-02499-s001.zip › pharmaceutics-2597105-supplementary.pdf]

Supplementary Materials

Cocrystal of ubiquinol: improved stability and bioavailability

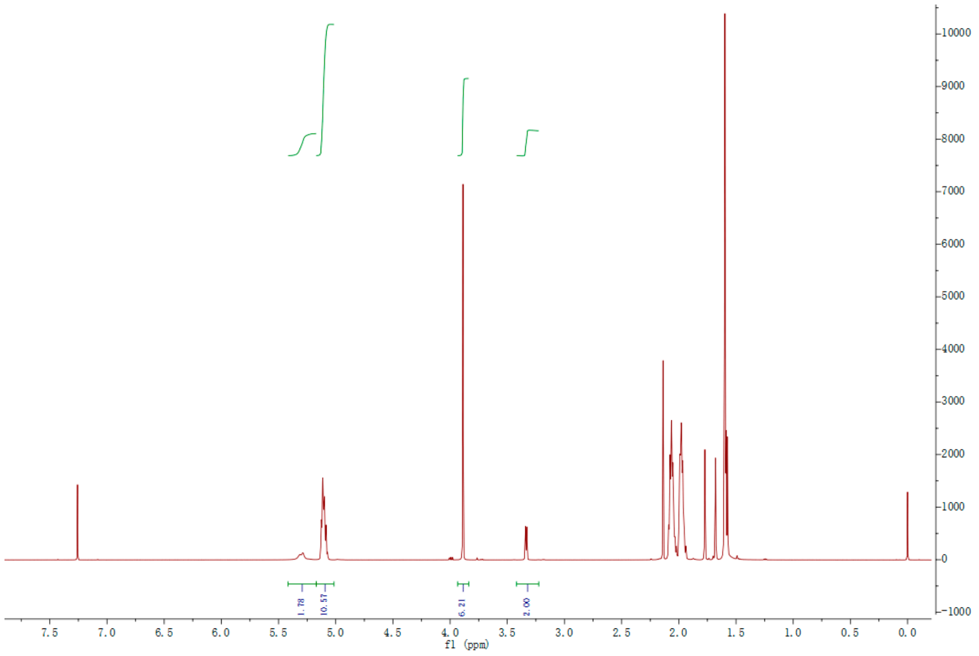

Figure S1. ^{1}H NMR spectra of ubiquinol.

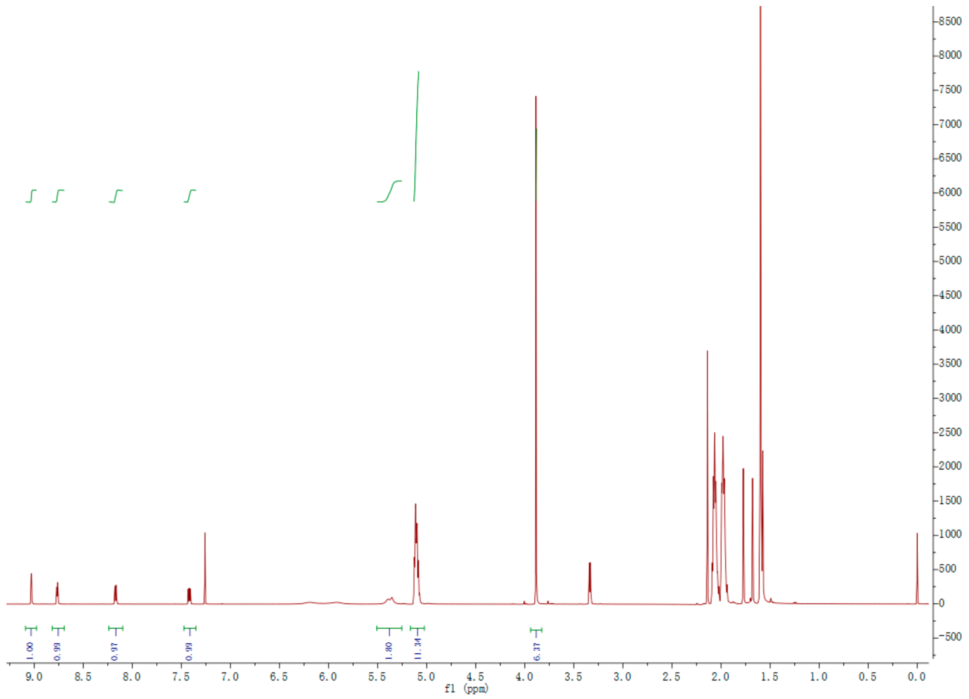

Figure S2. ^{1}H NMR spectra of UQ-NC cocrystal.

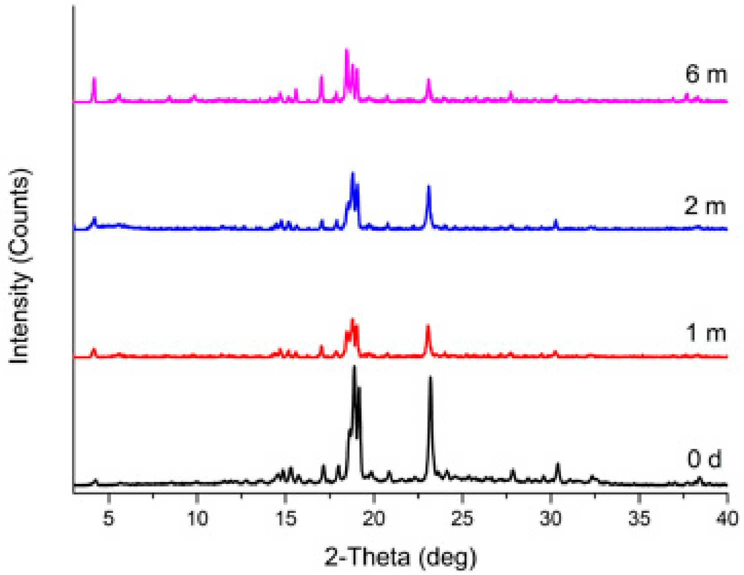

(b)

FigureS3. XRPD pattern of UQ-NC cocrystal during storage at 40℃/75% RH.

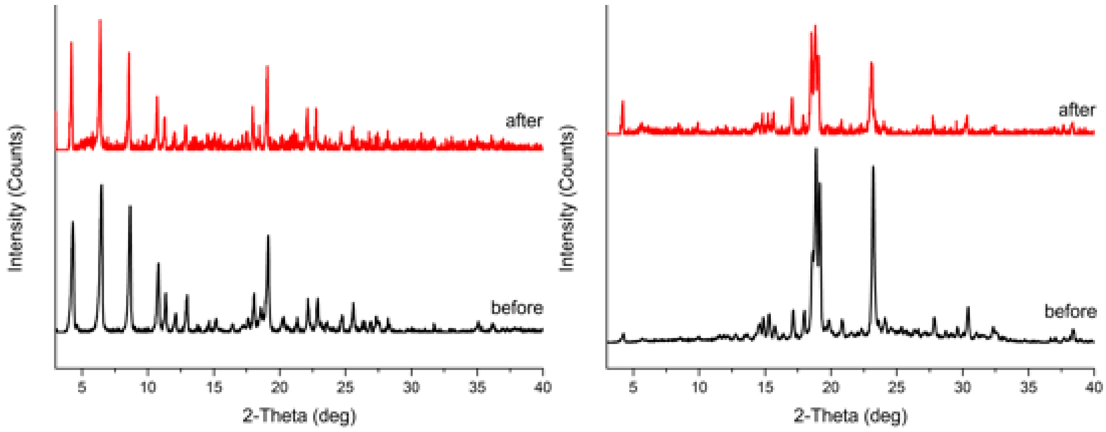

(a) (b)

Figure S4. XRPD patterns before and after dissolution test (SGF) (a) ubiquinol (b) UQ-NC cocrystal.

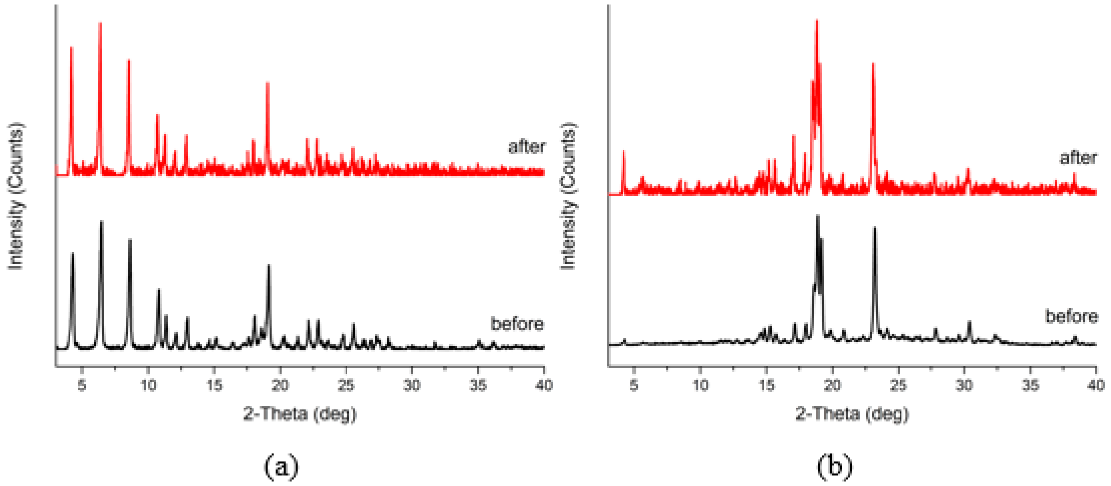

Figure S5. XRPD patterns before and after dissolution test (SIF) (a) ubiquinol (b) UQ-NC cocrystal
